# Shared Decision-Making for Prostate Cancer Screening Among Minority Men in the United States: A Systematic Review

**DOI:** 10.1093/geroni/igaf122.4191

**Published:** 2025-12-31

**Authors:** Hyesong Joung, Hyeyeon Shin, Randy Jones

**Affiliations:** The University of Virginia, Charlottesville, Virginia, United States; The Ohio State University, Columbus, Ohio, United States; The University of Virginia, Charlottesville, Virginia, United States

## Abstract

Prostate cancer screening (PCS) remains controversial in its efficacy, making shared decision-making (SDM) essential to ensure informed choices. However, racial and ethnic minority men in the United States experience persistent disparities in PCS participation and outcomes. Despite these disparities, how SDM interventions have been designed, implemented, and evaluated for these populations remains unclear. To address this gap, this systematic review examined SDM interventions for PCS among minority men, with a specific focus on the extent to which interventions incorporated key SDM features (information exchange, deliberation, and decision implementation) based on the SDM framework. Six databases (PubMed, Embase, CINAHL, PsycINFO, Web of Science, and Scopus) were searched for U.S.-based, English-language studies published in the past ten years that included minority participants comprising more than 30% of the sample. Eighteen studies met the inclusion criteria. Most targeted African American men, and the majority of interventions were delivered as single-session programs. Participant age criteria were inconsistent across studies. While all studies supported information exchange, few incorporated the full spectrum of SDM components (deliberation and decision implementation). Knowledge improvement was consistently reported, but effects on decision quality and screening behavior remain unclear. These findings highlight challenges in both the design and evaluation of SDM interventions, including limited application of core SDM features and underrepresentation of diverse minority populations. This review calls for future studies to develop and test consistent, comprehensive SDM interventions. Such efforts are needed to advance research on informed, preference-sensitive screening decisions and ultimately contribute to more equitable, patient-centered prostate cancer care.

